# Artificial Intelligence Technologies for Sign Language

**DOI:** 10.3390/s21175843

**Published:** 2021-08-30

**Authors:** Ilias Papastratis, Christos Chatzikonstantinou, Dimitrios Konstantinidis, Kosmas Dimitropoulos, Petros Daras

**Affiliations:** Visual Computing Lab, Information Technologies Institute (ITI), Centre for Research and Technology Hellas (CERTH), 57001 Thessaloniki, Greece; chatziko@iti.gr (C.C.); dikonsta@iti.gr (D.K.); dimitrop@iti.gr (K.D.); daras@iti.gr (P.D.)

**Keywords:** sign language recognition, sign language representation, sign language capturing, applications

## Abstract

AI technologies can play an important role in breaking down the communication barriers of deaf or hearing-impaired people with other communities, contributing significantly to their social inclusion. Recent advances in both sensing technologies and AI algorithms have paved the way for the development of various applications aiming at fulfilling the needs of deaf and hearing-impaired communities. To this end, this survey aims to provide a comprehensive review of state-of-the-art methods in sign language capturing, recognition, translation and representation, pinpointing their advantages and limitations. In addition, the survey presents a number of applications, while it discusses the main challenges in the field of sign language technologies. Future research direction are also proposed in order to assist prospective researchers towards further advancing the field.

## 1. Introduction

Sign language (SL) is the main means of communication between hearing-impaired people and other communities and it is expressed through manual (i.e., body and hand motions) and non-manual (i.e., facial expressions) features. These features are combined together to form utterances that convey the meaning of words or sentences [1]. Being able to capture and understand the relation between utterances and words is crucial for the Deaf community in order to guide us to an era where the translation between utterances and words can be achieved automatically [2]. The research community has long identified the need for developing sign language technologies to facilitate the communication and social inclusion of hearing-impaired people. Although the development of such technologies can be really challenging due to the existence of numerous sign languages and the lack of large annotated datasets, the recent advances in AI and machine learning have played a significant role towards automating and enhancing such technologies.

Sign language technologies cover a wide spectrum, ranging from the capturing of signs to their realistic representation in order to facilitate the communication between hearing-impaired people, as well as the communication between hearing-impaired and speaking people. More specifically, sign language capturing involves the accurate extraction of body, hand and mouth expressions using appropriate sensing devices in marker-less or marker-based setups. The accuracy of sign language capturing technologies is currently limited by the resolution and discrimination ability of sensors and the fact that occlusions and fast hand movements pose significant challenges to the accurate capturing of signs. Sign language recognition (SLR) involves the development of powerful machine learning algorithms to robustly classify human articulations to isolated signs or continuous sentences. Current limitations in SLR lie in the lack of large annotated datasets that greatly affect the accuracy and generalization ability of SLR methods, as well as the difficulty in identifying sign boundaries in continuous SLR scenarios.

On the other hand, sign language translation (SLT) involves the translation between different sign languages, as well as the translation between sign and speaking languages. SLT methods employ sequence-based machine learning algorithms and aim to bridge the communication gap between people signing or speaking different languages. The difficulties in SLT lie in the lack of multilingual sign language datasets, as well as the inaccuracies of SLR methods, considering that the gloss recognition (performed by SLR methods) is the initial step of the SLT methods . Finally, sign language representation involves the accurate representation and reproduction of signs using realistic avatars or signed video approaches. Currently, avatar movements are deemed unnatural and hard to understand by the Deaf community due to inaccuracies in skeletal pose capturing and the lack of life-like features in the appearance of avatars.

**Figure 1:**
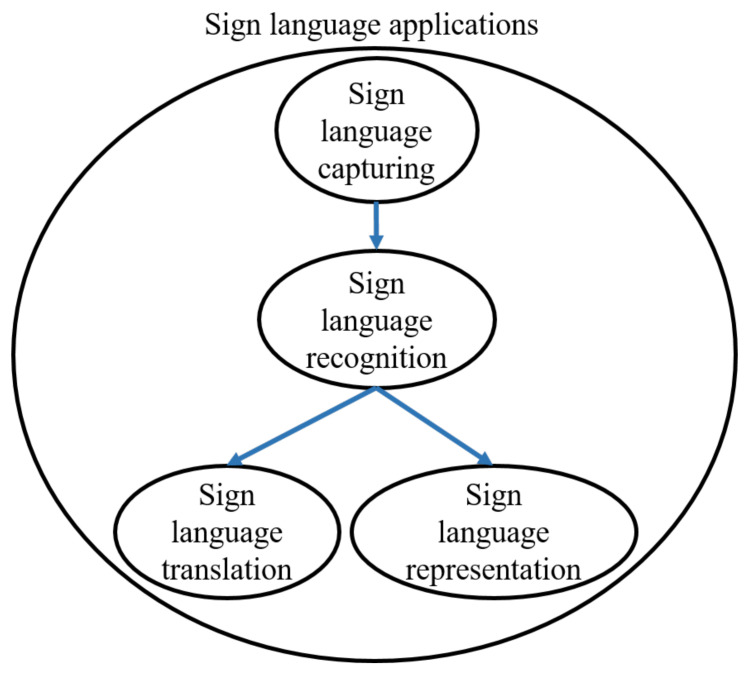
Sign language technologies.

Sign language technologies are connected in a way that affect each other as seen in Figure 1. The accurate extraction of hand and body motions as well as facial expressions plays a crucial role to the success of the machine learning algorithms that are responsible for the robust recognition of signs. Moreover, the accurate sign language recognition significantly affects the performance of sign language translation and representation methods. The breakthroughs in sensorial devices and AI have paved the way for the development of sign language applications that can immensely facilitate hearing-impaired people in their everyday life.

Previous literature reviews mainly concentrate on specific sign language technologies, such as video-based and sensor-based sign language recognition [3,4,5,6,7] and sign language translation [8,9]. Lately, with the development of sign language applications, there are also reviews that presented sign language systems to facilitate hearing-impaired people in teaching and learning, as well as in voice and text interpretation systems [10,11]. However, there is no systematic review that presents all sign language technologies and their relations with each other. This review aims to fill this gap by presenting the advances of AI in all sign language technologies, ranging from capturing and recognition to translation and representation and concludes by describing recent sign language applications that can considerably facilitate the communication among hearing-impaired and speaking people. The main purpose of this review is to demonstrate the importance of using AI technologies in sign language to facilitate deaf and hearing-impaired people in their communication with other communities. In addition, this review aims at familiarizing researchers with the state-of-the-art in all sign language technologies and propose future research directions that can facilitate the development of even more accurate approaches that can lead to mainstream products for the Deaf community. More specifically, the objectives of this review can be summarized as follows:

- A comprehensive overview of the use of AI technologies in various sign language tasks (i.e., capturing, recognition, translation and representation), along with their importance to their field, is provided.
- The advantages and limitations of modern sign language technologies and the relations between them are discussed and explored.
- Possible future directions in the development of AI technologies for sign language are suggested to facilitate prospective researchers in the field.

The rest of this survey is organized as follows. In Section 2, the literature search guideline is presented. Sign language capturing sensors are described in Section 3. In Section 4, sign language recognition methods are categorized and discussed. Sign language representation approaches and applications are presented in Section 5 and Section 6, respectively. Finally, conclusions and potential future research directions are highlighted in Section 7.

## 2. Literature Search

A systematic literature search was performed by adopting the PRISMA guidelines [12]. The articles were extracted in June 2021 from three academic databases, namely Scopus (https://www.scopus.com/home.uri), (link, accessed on 28 May 2021), ProQuest (https://www.proquest.com/), (link, accessed on 28 May 2021) and IeeeXplore (https://ieeexplore.ieee.org/Xplore/home.jsp), (link, accessed on 28 May 2021). The articles that were not peer-reviewed or written in English were discarded. Since this review deals with AI technologies for sign language, the search was based on the following condition:

TITLE-ABSTRACT-KEYWORDS ( sign AND language AND ( recognition OR application(*) OR avatar(*) OR representation(*) OR translation OR captur(*) OR generation OR production ) ) AND PUBLISH YEAR > 2018 AND ( LIMIT-TO ( DOCTYPE , "ar" ) OR LIMIT-TO ( DOCTYPE , "cp" ) OR LIMIT-TO ( DOCTYPE , "ch" ) ) AND ( LIMIT-TO ( LANGUAGE , "English" ) ) AND ( LIMIT-TO ( PUBSTAGE , "final" ) ) AND ( LIMIT-TO ( SUBJAREA , "COMP" ) OR LIMIT-TO ( SUBJAREA , "ENGI" ) )

The aforementioned search condition describes the existence of the above words (i.e., recognition, translation, etc.) in the title, abstract or keywords of the literature works. In this context, (*) allows for variations in the search terms (i.e., captur(*) allows the existence of words, such as capture, capturing, etc.). In addition, the search is performed for papers published after 2018 since the field is evolving with fast pace and older methods are rendered quickly obsolete. To this end, this review aims to present only the latest and best works related to sign language technologies. Finally, the papers included in this review have been published as journal articles, conference proceedings and book chapters (i.e., DOCTYPE) in the fields of computing and engineering (i.e., SUBJAREA).

**Figure 2:**
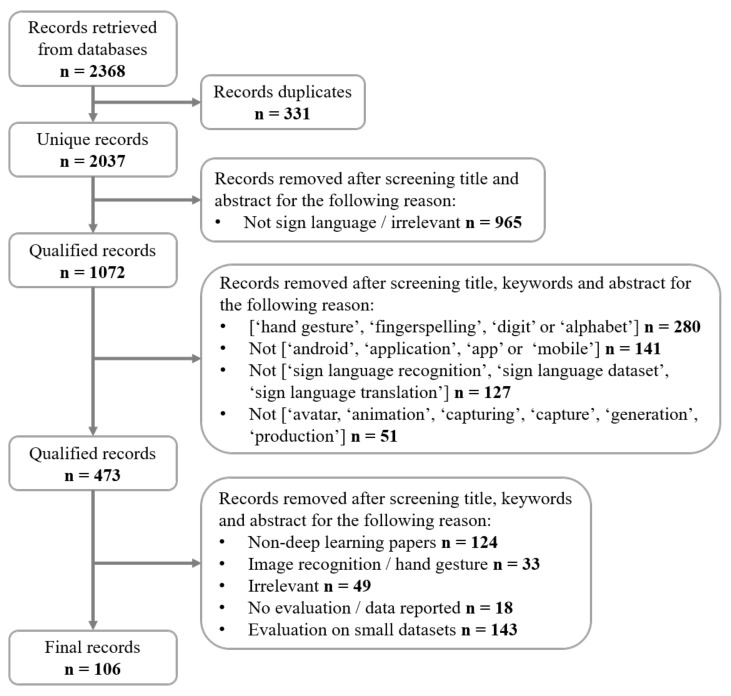
Flowchart of the systematic literature search process.

The number of the records retrieved from the three databases is 2368. From this number, 331 duplicate records are removed, leading to 2037 unique records. After screening title, abstract and finally the full text with various criteria to discard irrelevant records, 106 records remain and are included in this review. The selection procedure is depicted in Figure 2.

## 3. Sign Language Capturing

Sign language capturing involves the recording of sign gestures using appropriate sensor setups. The purpose is to capture discriminative information from the signs that will allow the study, recognition and 3D representation of signs at later stages. Moreover, sign language capturing enables the construction of large datasets that can be used to accurately train and evaluate machine learning sign language recognition and translation algorithms.

### 3.1. Capturing Sensors

The most common means of recording sign gestures is through visual sensors that are able to capture fine-grained information, such as facial expressions and body postures, that is crucial for understanding sign language. Cerna et al. in [13] employed a Kinect sensor [14] to simultaneously capture red-green-blue (RGB) image, depth and skeletal information towards the recording of a multimodal dataset with Brazilian sign language. Similarly, Kosmopoulos et al. in [15] captured realistic real-life scenarios with sign language using the Kinect sensor. The dataset contains isolated and continuous sign language recordings with RGB, depth and skeletal information, along with annotated hand and facial features. Contrary to the previous methods that use a single Kinect sensor, this work additionally employs a machine vision camera, along with a television screen, for sign demonstration. Sincan et al. in [16], captured isolated Turkish sign language glosses using Kinect sensors with a large variety of indoor and outdoor backgrounds, revealing the importance of capturing videos with various backgrounds. Adaloglou et al. in [17], created a large sign language dataset with RealSense D435 sensor that records both RGB and depth information. The dataset contain continuous and isolated sign videos and is appropriate for both isolated and continuous sign language recognition tasks.

Another sensor that has been employed for sign language capturing is Leap Motion, which has the ability to capture 3D positions of hand and fingers at the expense of having to operate close to the subject. Mittal et al. in [18], employed this type of sensor to record sign language gestures. Other setups with antennas and readers of radio-frequency identification (RFID) signals have also been adopted for sign language recognition. Meng et al. in [19], extracted phase characteristics of RFID signals to detect and recognize sign gestures. The training setup consists of an RFID reader, an RFID tag and a directional antenna. The recorded human should stand between the reader and the tag for a proper capturing. Moreover, the recognition system is signer-dependent.

On the other hand, wearable sensors have been adopted for capturing sign language gestures. Galea et al. in [20], used electromyography (EMG) to capture electrical activity that was produced during arm movement. The Thalmic MYO armband device was used for the recording of Irish sign language alphabet. Similarly, Zhang et al. [21] used a wearable device to capture EMG and inertial measurement unit (IMU) signals, while they used a convolutional neural network (CNN) [22] followed by a long short-term memory (LSTM) [23] architecture to recognize American sign language at both word and sentence levels. One disadvantage of the method is that its performance has not been evaluated under walking condition. Hou et al. in [24], proposed Sign-Speaker, which was deployed on a smartwatch to collect sign signals. Then, these signals were sent to a smartphone and were translated into spoken language in real-time. In this method, a very simple capturing setup is required, consisting of a smartwatch and a smartphone. However, their system recognizes a limited number of signs and it cannot generalize well to new users. Wang et al. in [25], employed a system with two armbands using both IMU and EMG sensors in order to capture fine-grained finger and hand positions and movements. How et al. in [26], used a low-cost dataglove with IMU sensors to capture sign gestures that were transmitted through Bluetooth to a smartphone device. Nevertheless, the employment of a single right-hand dataglove limited the number of signs that could be performed by this setup.

Each of the aforementioned sensor setups for sign language capturing has different characteristics, which makes it suitable for different applications. Kinect sensors provide high resolution RGB and depth information but their accuracy is restricted by the distance from the sensors. Leap Motion also requires a small distance between the sensor and the subject, but their low computational requirements enable its usage in real-time applications. Multi-camera setups are capable of providing highly accurate results at the expense of increased complexity and computational requirements. A myo armband that can detect EMG and inertial signals is also used in few works but the inertial signals may be distorted by body motions when people are walking. Smartwatches are really popular nowadays and they can also be used for sign language capturing but their output can be quite noisy due to unexpected body movements. Finally, datagloves can provide highly accurate sign language capturing results in real-time. However, the tuning of its components (i.e., flex sensor, accelerometer, gyroscope) may require a trial and error process that is impractical and time-consuming. In addition, signers tend to not prefer datagloves for sign language capturing as they are considered invasive.

### 3.2. Datasets

Datasets are crucial for the performance of methodologies regarding sign language recognition, translation and synthesis and as a result a lot of attention has been drawn towards the accurate capturing of signs and their meticulous annotation. The majority of the existing publicly available datasets are captured with visual sensors and are presented below.

#### 3.2.1. Continuous Sign Language Recognition Datasets

Continuous sign language recognition (CSLR) datasets contain videos of sequences of signs instead of individual signs and are more suitable for developing real-life applications. Phoenix-2014 [27] is one of the most popular CSLR dataset with recordings of weather forecasts in German sign language. All videos were recorded with 9 signers at a frame rate of 25 frames per second. The dictionary has 1081 unique glosses and the dataset contains 5672 videos for training, 540 videos for validation and 629 videos for testing. The same authors created an updated version of Phoenix-2014, called Phoenix-2014-T [28], with spoken language translations, which makes it appropriate for both CSLR and sign language translation experiments. It contains 8257 videos from 9 different signers performing 1088 unique signs and 2887 unique words. Although all recordings are performed in a controlled environment, Phoenix-2014 and Phoenix-2014-T are both challenging datasets with large vocabularies and varying number of samples per sign with a few signs having a single sample. Similarly, BSL-1K [29] contains video recordings from British news broadcasts, along with automatically extracted annotations from provided subtitles. It is a large database with 273,000 samples from 40 signers that is also used for sign language segmentation. Another notable dataset is CSL [30,31] that contains Chinese words widely used in daily communication. The dataset has 100 sentences with signs that were performed from 50 signers. The recordings are performed in a lab with predefined conditions (i.e., background, lighting). The vocabulary size is 178 words that are performed multiple times, resulting in high recognition results achieved by SLR methods. GRSL [15] is another CSLR dataset of Greek sign language that is used in home care services, which contains multiple modalities, such as RGB, depth and skeletal joints. On the other hand, GSL [17] is a large Greek sign language dataset created to assist communication of Deaf people with public service employees. The dataset was created with a RealSense D435 sensor that records both RGB and depth information. Furthermore, it contains both continuous and isolated sign videos from 15 predefined scenarios. It is recorded on a laboratory environment, where each scenario is repeated five consecutive times.

#### 3.2.2. Isolated Sign Language Recognition Datasets

Isolated sign language recognition (ISLR) datasets are important for identifying and learning discriminative features for sign language recognition. CSL-500 [31,32] is the isolated version of CSL but it contains 500 unique glosses performed from the same 50 signers. CSLR methods usually adopt this dataset for feature learning prior to finetuning on the CSL dataset. MS-ASL [33] is another widely employed ISLR dataset with 1000 unique American sign language glosses. It contains recordings collected from YouTube platform from 222 signers with a large variance in background settings, which makes this dataset suitable for training complex methods with strong representation capabilities. Similarly, WASL [34] is an ISLR dataset with 2000 unique American sign glosses performed by 119 signers. The videos have different background and illumination conditions, which makes it a challenging ISLR benchmark dataset. On the other hand, AUTSL is a Turkish sign language dataset captured under various indoor and outdoor backgrounds, while LSA64 [35] is an Argentinian sign language dataset that includes 3200 videos, in which 10 non-expert subjects execute 5 repetitions of 64 different types of signs. LSA64 is a small and relatively easy dataset, where SLR methods achieve outstanding recognition performance. Finally, IsoGD [36] is a gesture recognition dataset that consists of 47,933 RGB-D videos performed by 21 different individuals and contains 249 gesture labels. Although IsoGD is a gesture recognition dataset, its large size and challenging illumination and background conditions allows the training of highly accurate ISLR methods.

#### 3.2.3. Discussion

A discussion about the aforementioned datasets can be made at this stage, while a detailed overview of the dataset characteristics is provided on Table 1. It can be seen that over time datasets become larger in size (i.e., number of samples) with more signers involved in them, as well as contain high resolution videos captured under various and challenging illumination and background conditions. Moreover, new datasets usually include different modalities (i.e., RGB, depth and skeleton). Recording sign language videos using many signers is very important, since each person performs signs with different speed, body posture and face expression. Moreover, high resolution videos capture more clearly small but important details, such as finger movements and face expressions, which are crucial cues for sign language understanding. Datasets with videos captured under different conditions enable deep networks to extract highly discriminative features for sign language classification. As a result, methodologies trained in such datasets can obtain greatly enhanced representation and generalization capabilities and achieve high recognition performances. Furthermore, although RGB information is the predominant modality used for sign language recognition, additional modalities, such as skeleton and depth information, can provide complementary information to the RGB modality and significantly improve the performance of SLR methods.

## 4. Sign Language Recognition

Sign language recognition (SLR) is the task of recognizing sign language glosses from video streams. It is a very important research area since it can bridge the communication gap between hearing and Deaf people, facilitating the social inclusion of hearing-impaired people. Moreover, sign language recognition can be classified into isolated and continuous based on whether the video streams contain an isolated gloss or a gloss sequence that corresponds to a sentence.

### 4.1. Continuous Sign Language Recognition

Continuous Sign Language Recognition aims at classifying signed videos to entire sentences (i.e., ordered sequence of glosses). CSLR is a very challenging task as it requires the recognition of glosses from video sequences without any knowledge of the sign boundaries (i.e., lack of ground truth annotations regarding the start and end of glosses). Most works adopt 2D or 3D-CNNs for feature extraction followed by temporal convolutional networks or recurrent neural networks (RNNs) for sequential information modelling. To measure CSLR performance, word error rate (WER) [38] is commonly adopted. WER measures the number of operations (i.e., substitutions, deletions and insertions) required to transform the predicted sequence into the target sequence.

Cui et al. [39] adopted a 2D-CNN followed by temporal 1D convolutional layers for feature extraction. The extracted spatio-temporal features were fed to a bidirectional long short-term memory (BLSTM) network for modelling the context of the entire sequence. The feature extractor was extended with a classifier and trained in a fully-supervised setting on isolated glosses for video to gloss alignment, while the BLSTM was used for CSLR. This two-step optimization process was conducted iteratively with Connectionist Temporal Classification (CTC) [40] and Cross-Entropy losses, until the network converged. Besides, the recognition model fused RGB with optical flow modalities and achieved a WER of 22.8% on the Phoenix-2014 dataset. Similarly, Koishybay et al. in [41], adopted a residual 2D-CNN with cascaded 1D convolutional layers for feature extraction, while for CSLR experiments, BLSTM was utilized. Their method generated gloss-level alignments using the Levenshtein distance in order to fine-tune the feature extractor. However, the authors stated that during the early iterations the model predicted poor alignment proposals, which hinders the training process and requires several iterations to converge. Cheng et al. in [42], proposed a 2D fully convolutional network with a feature enhancement module that did not require iterative training. Instead, it provided extra supervision and assisted the CSLR network to learn better gloss alignments. Niu et al. in [43], proposed a 2D-CNN followed by a Transformer network for CSLR. They used three stochastic methods to drop frames of the input video, to randomly stop gradients of back-propagation and to model glosses using hidden states, respectively, which led to better CSLR performance. Nevertheless, the randomness ratio of these stochastic processes must be tuned carefully to achieve good recognition rates. Generally, CSLR methods based on 2D-CNNs achieve great recognition performance. More specifically, 2D-CNNs extract descriptive features from the frame sequences, while the sequence modelling mechanisms align efficiently the input video and the output predictions. However, they usually require complex training strategies, such as iterative optimization techniques, to achieve strong feature extraction capabilities.

On the other hand, some works chose to incorporate attention mechanisms for CSLR. Pan et al. in [44], used a key-frame sampling technique to extract the most descriptive frames of the video. Then, a vector representation was constructed from the skeletal data of the key-frames, which was fed to an attention-based BLSTM to model the temporal information. Huang et al. [45] proposed an adaptive encoder-decoder architecture to learn the temporal boundaries of the video. Furthermore, a hierarchical BLSTM with attention over sliding windows was used on the decoder to weigh the importance of the input frames. Li et al. in [46], used a pyramid structure of BLSTMs in order to find key actions of the video representations, which were produced from the 2D-CNN. Moreover, an attention-based LSTM was used to align the input and output sequences and the whole network was trained jointly with Cross-Entropy and CTC losses.

Recently, the self-attention mechanism has been introduced in a variety of models, such as the Transformer, and has also been adopted by CSLR methods. Slimane et al. in [47], proposed two data streams with cropped hand images and full images. The two modalities were passed through two 2D-CNNs to extract the spatial features. Then, the modalities were synchronized by a self-attention module to obtain better contextual information and generate efficient video representations for CSLR. Zhou et al. [48], adopted a fully-inception architecture with 2D and 1D convolutional layers along with a self-attention to further improve the feature extraction capabilities of the inception layers.

Reinforcement techniques have also been applied for CSLR, along with Transformer networks. Zhang et al. in [49], adopted a 3D-CNN followed by a Transformer network that was responsible for recognizing gloss sequences from input videos. Instead of training the model with cross-entropy loss, they used the REINFORCE algorithm [50] to directly optimize the model by using WER as the reward function of the agent (i.e., the feature extractor). Wei et al. in [51], used a semantic boundary detection algorithm with reinforcement learning to improve CSLR performance. A spatio-temporal feature extractor learned the video representations. Then, the detection algorithm used reinforcement learning to detect gloss timestamps from video sequences and refine the final video representations. The evaluation metric was used again as the reward function. The major limitation of this method is the need for a careful selection of the pooling size, which defines the action search space for the reinforcement learning agent.

Papastratis et al. [52] constructed a cross-modal approach in order to effectively model intra-gloss dependencies by leveraging information from text. This method extracted video features using a video encoder that consisted of a 2D-CNN followed by temporal convolutions and a BLSTM, while text representations were obtained from an LSTM. Finally, these embeddings were aligned in a joint latent space. The improved representations led to great CSLR performance, achieving WERs of 24.0% and 3.52% on Phoenix-2014 and GSL SI, respectively. Papastratis et al. in their latest work [53], employed a generative adversarial network to evaluate the predictions of the video encoder. In addition, contextual information was incorporated to improve recognition performance on sign language conversations.

Due to their efficient feature extraction capabilities, 3D-CNNs have also been adopted by many researchers for CSLR. Wei et al. in [54], used a 3D residual CNN along with a BLSTM, while they applied grammatical rules sign language. The text was split into isolated words and *n*-grams, which are modelled using two classifiers. The two classifiers aimed to recognize each word independently and based on the context in contrast to CTC, which models the whole sequence. Pu et al. in [55], employed a 3D-CNN with an LSTM decoder and a CTC decoder that were jointly aligned with a soft dynamic time warping (soft-DTW) [56] alignment constraint. The network was trained recursively with the proposed alignments from soft-DTW. The method achieved WERs of 6.1% and 32.7% on CSL Split 1 and CSL Split 2, respectively. Guo et al. in [57], developed a fully convolutional approach with a 3D-CNN followed by 1D temporal convolutional layers. The 1D CNN block had a hierarchical structure with small and large receptive fields to capture short- and long-term correlations in the video, while the entire architecture was trained with CTC loss. 3D-CNNs are computationally expensive methods that require pre-training on large-scale datasets and cannot be tuned directly for CSLR. To this end, sliding window techniques are adopted to create informative features. To tackle this problem, some works incorporated pseudo-labelling, which is an optimization process that adds predicted labels on the training set. Pei et al. in [58], trained a deep 3D-CNN with CTC and generate clip-level pseudo-labels from the alignment of CTC to obtain better feature representations. To improve the quality of pseudo-labels, Zhou et al. in [59], proposed a dynamic decoding method instead of greedy decoding to find better alignment paths and filter out the wrong pseudo-labels. Their method applied the I3D [60] network from the action recognition field along with temporal convolutions and bidirectional gated recurrent units (BGRU) [61]. Moreover, the proposed method achieved a WER of 34.5% on the Phoenix-2014 dataset. However, pseudo-labelling required many iterations, while initial labels affected the convergence of the optimization process.

In Table 2, several methods are compared on the test set of the most commonly adopted datasets for continuous sign language recognition. From the experimental results it is shown that multi-modal methods achieve the lowest WERs. More specifically, STMC [62] has the best recognition rates on Phoenix-2014, CSL Split 1 and CSL Split 2 datasets using RGB, hands and skeleton modalities, while SLRGAN [53], employing the RGB and text modality, achieves superior performance on the GSL SI and GSL SD datasets.

### 4.2. Isolated Sign Language Recognition

Isolated sign language recognition refers to the task of accurately detecting single sign gestures from videos and thus it is usually tackled similar to action and gesture recognition, as well as other types of video processing and classification tasks with the extraction and learning of highly discriminative features [63,64,65]. In the literature, a common approach to the task of isolated sign language recognition is the extraction of hand and mouth regions from the video sequences in an attempt to remove noisy backgrounds that can inhibit classification performance. Liao et al. in [66], proposed a video-based SLR method that was based on hand region extraction and classification using 3D ResNet networks and BLSTM layers. Similarly, Aly et al. in [67], developed an ISLR method that segmented hand regions from images using DeepLabv3+ algorithm [68], extracted features from these regions using a Convolutional Self-Organizing Map and classified the features using a deep recurrent neural network consisting of 3 BLSTM layers. Gökçe et al. in [69], proposed 3D-CNN networks for the processing of hand, upper body and face image regions and the fusion of these streams in the score level to accurately classify isolated signs. The authors stated that their method performs comparatively worse on mono-morphemic signs performed with a single hand, rather than on temporally more complex signs with two-handed gestures. On the other hand, Zhang et al. in [70], proposed the Multiple extraction and Multiple prediction (MEMP) network that consists of alternating 3D-CNN networks and Convolutional LSTM layers that extracted spatio-temporal features from video sequences multiple times, enabling the network to achieve 99.06% and 78.85% accuracy in the LSA64 and IsoGD datasets, respectively. Li et al. in [71], proposed a SLR method that was based on the transferring of cross-domain knowledge of news signs to a base model and improve its performance using domain-invariant features.

To further improve the accuracy and robustness of SLR methods, several researchers proposed the extraction of other types of features, such as optical flow and skeletal joints from visual cues. These multi-stream networks are more computationally expensive than their single stream counterparts, but they have the advantage of overcoming confusing cases regularly met when a single type of features is employed. Sarhan et al. in [72], proposed a two-stream network architecture that received as input RGB and optical flow data, extracted features using I3D networks and performed late fusion at the score level for accurate sign language recognition. Rastgoo et al. in [73], proposed a multi-stream SLR method that utilized as input hand image regions, hand heatmaps and 2D projections of hand skeletal joints to images. These input data were processed using 3D-CNN networks, concatenated and fed to LSTM layers for sign recognition. Konstantinidis et al. in [74], proposed a SLR methodology that was based on the processing and late fusion of body and hand skeletal features using LSTM layers. Apart from the raw joint coordinates, the authors also utilized joint-line distances, which led to a significant improvement in the performance of the method, reaching 98.09% accuracy in the LSA64 dataset. In a later work [75], the same authors introduced additional streams that processed RGB video sequences and optical flow data, enhancing even more the performance of their method, ultimately achieving 99.84% accuracy in the LSA64 dataset. Similarly, Papadimitriou et al. in [76], proposed a multi-stream SLR method that processes hand and mouth regions, as well as optical flow and skeletal features for the accurate classification of signs. These features were concatenated and fed to a temporal deformable convolutional attention-based encoder-decoder that predicts the sign class. Gündüz et al. in [77], employed a multi-stream SLR approach that received as input RGB video sequences, optical flow sequences and body and hand skeletal features and performed a late fusion to accurately classify Turkish signs. Bilge et al. in [78], proposed a SLR method that can generalize well on unseen signs. To achieve this, the authors employed two 3D-CNN networks followed by BLSTM layers for the extraction of short-term and long-term feature representations from body and hand video sequences. In addition, the authors employed a BERT model [79] for the extraction of textual sign representations from text descriptions of how the signs were performed. Finally, they used a bi-linear compatibility function to associate video and text representations.

In an effort to derive more discriminative features, Rastgoo et al. in [63], proposed a multi-stream SLR method that gets as input hand regions, 3D hand pose features and Extra Spatial Hand Relation features (i.e., orientation and slope of hands). These features were concatenated and fed to an LSTM layer to derive the sign class. In this way, the authors managed to achieve a really high accuracy of 86.32% in the challenging IsoGD dataset. Kumar et al. in [64], proposed Spatial 3D Relational Features for sign language recognition. These features were computed from the area and perimeter of polygons formed by quadruples of skeletal joints. Then, the class of a test sign was predicted by comparing the sign with the training set using global alignment kernels. In another work [80], Kumar et al. introduced two novel features for accurate sign language recognition that were named colour-coded topographical descriptors. These descriptors were formed as images from the computation of joint distances and angles. Finally, these descriptors were processed by 2D CNNs and merged to derive the class of the sign.

Recently, the advances in deep learning led several isolated SLR methods to leverage attention mechanisms, transformer networks and graph convolutional networks. Attention mechanisms in particular enable a deep network to pay more attention on features that are important for a classification task and are widely employed by most state-of-the-art SLR methods. Parelli et al. in [81], proposed a multi-stream SLR method that processes hand and mouth image regions as well as 3D hand skeletal data. All streams were concatenated and fed to an attention CNN network that accurately predicts the class of the sign. Attention LSTM, attention GRU and Transformer networks were also tested but they led to inferior performance. De Amorim et al. in [82], proposed an American SLR method that extracts skeletal data from video sequences and then processes them using a Spatio-Temporal Graph Convolutional Network (GCN) [83]. Tunga et al. in [84], proposed a SLR method that extracts skeletal features from video sequences and then employs a GCN network to model spatial dependencies among the skeletal data, as well as a BERT model to model temporal dependencies among the skeletal data. The two representations were finally merged to derive the class of the sign. A limitation of this approach is that the model cannot differentiate in-plane and out-of-plane movements due to the use of only 2D spatial information. In a similar fashion, Meng et al. in [85], proposed a GCN with multi-scale attention modules to process the extracted skeletal data and model their long-term spatial and temporal dependencies. In this way, the authors achieved a really high accuracy of 97.36% in the CSL-500 dataset. GCNs are computationally lighter than the image processing networks, but they often cannot extract highly enriched features, thus leading to inferior performance, as noted in [82].

Finally, the wide adoption of RGB-D sensors for action and gesture recognition has led several researchers to adopt them for multi-modal sign language recognition as well. However, the performance of such multi-modal methodologies is currently limited by the small number of large publicly available RGB-D datasets and the mediocre accuracy of depth information. Tur et al. in [86], proposed a Siamese deep network for the concurrent processing of RGB and depth sequences. The extracted features were then concatenated and passed to an LSTM layer for isolated sign language recognition. Ravi et al. in [87], proposed a multi-modal SLR method that was based on the processing of RGB, depth and optical flow sequences. Each stream employed CNN layers to process the sequences and then, all features were fused together and fed to a CNN model for classification. Rastgoo et al. in [88], proposed a multi-modal SLR method that leverages RGB and depth video sequences to achieve an accuracy of 86.1% in the IsoGD dataset. More specifically, the authors extracted pixel-level, optical flow, deep hand and hand pose features for each modality, concatenated these features across both modalities and classified them to sign classes using an LSTM layer. The authors stated that there were signs with similar appearance and motion features that led to misclassification errors and thus they proposed the use of augmentation strategies, high capacity networks and more data samples.

Huang et al. in [89], proposed the use of RGB, depth and skeletal data as input to attention-based 3D-CNNs and attention-based BLSTMs in order for the proposed SLR method to pay attention to spatio-temporal dependencies in the input data and fuse the input streams in an optimal way. Huang et al. in [90], proposed a sequence-to-sequence approach that detects key frames to remove noisy information from video sequences. Then, they extracted CNN features from these key frames, histogram-of-gradients (HOG) features from depth motion maps and trajectory features from skeletal data. These features were finally concatenated and fed to an encoder-decoder LSTM network that predicted sub-words that form the signed word. Zhang et al. in [91], proposed a highly accurate SLR method that initially selected pairs of aligned RGB-D images to reduce redundancy. Then, the proposed method computed discriminative features from hand regions using a spatial stream and extracted depth motion features using a temporal stream. Both streams were finally fused by a convolutional fusion layer and the output feature vector was used for classification. The authors reported that occlusions and the surface materials can significantly affect the quality of depth images, degrading the performance of their model. Common failure cases among most ISLR methodologies are the difficulty in differentiating signs when performed differently by users and the inability to accurately classify signs with similar hand shapes and positions. An overview of the performance of ISLR methods on well-known datasets are presented in Table 3.

### 4.3. Sign Language Translation

Sign Language Translation is the task of translating videos with sign language into spoken language by modeling not only the glosses but also the language structure and grammar. It is an important research area that facilitates the communication between the Deaf and other communities. Moreover, the SLT task is more challenging compared to CSLR due to the additional linguistic rules and the representation of spoken languages. SLT methods are usually evaluated using the bilingual evaluation understudy (BLEU) metric [92]. BLEU is a translation quality score that evaluates the correspondence between the predicted translation and the ground truth text. More specifically, BLEU-*n* measures the *n*-gram overlap between the output and the reference sentences. BLEU-1,2,3,4 are reported to provide a clear view of the actual translation performance of a method. Camgoz et al. in [28], adopted an attention-based neural machine translation architecture for SLT. The encoder consisted of a 2D-CNN and an LSTM network, while the decoder consists of word embeddings with an attention LSTM. The authors stated that the method is prone to errors when spoken words are not explicitly signed in the video but inferred from the context. Their method set the baseline performance on Phoenix-2014-T with a BLEU-4 score of 18.4. Orbay et al. in [93], compared different gloss tokenization methods using either 2D-CNN, 3D-CNN, LSTM or Transformer networks. In addition, they investigated the importance of using full frames compared to hand images as the first provide useful information regarding the face and arms of the signer for SLT. On the other hand, Ko et al. in [94], utilized human keypoints extracted from the video, which were then fed to a recurrent encoder-decoder network for sign language translation. Furthermore, the skeletal features were extracted with OpenPose and then normalized to improve the overall performance. Then, they were fed to the encoder, while the translation was generated from the attention decoder. Differently, Zheng et al. in [95], used a preprocessing algorithm to remove similar and redundant frames of the input video and increase the processing speed of the neural network without losing information. Then, they employed an SLT architecture that consisted of a 2D-CNN, temporal convolutional layers and bidirectional GRUs. Their method was able to deal with long videos that have long-term dependencies, improving the translation quality. Zhou et al. in [62], proposed a multi-modal framework for CSLR and SLT tasks. The proposed method used 2D-CNN, 1D convolutional layers and several BLSTMs and learned both spatial and temporal dependencies between different modalities. The proposed method achieved a BLEU-4 score of 23.65 on the test set of Phoenix-2014-T. However, due to the multi-modal cues, this method is very computationally heavy and requires several hours of training.

Recently, Transformer networks have also been employed for sign language translation due to their success in natural language processing tasks. Camgoz et al. in [96], introduced a joint architecture for CSLR and SLT with a Transformer encoder-decoder network. The network was trained with CTC and Cross-Entropy losses, while the gloss-level supervision improved the SLT performance. The authors evaluated various configurations of their method and stated that directly translating from video representations can improve the translation quality. A limitation of this approach was in translating numbers as there was no such context available during training. In their latest work, Camgoz et al. in [97], adopted additional modalities and a cross-modal attention to synchronize the different streams and model both inter- and intra-contextual information. Kim et al. in [98], used a deep neural network for human keypoint extraction that were fed to a transformer encoder-decoder network, while the keypoints were normalized based on the neck location. A comparison of existing methods for SLT that are evaluated on the Phoenix-2014-T dataset, is shown in Table 4. Overall, Transformer-based SLT methods achieve slightly better performance than RNN-based methods, which indicates the importance of attention mechanism for SLT. In addition, using multiple modalities can also improve the translation quality.

## 5. Sign Language Representation

The automatic and realistic sign language representation is vital for each sign language system. The representation of a sentence in sign language instead of a plain text can make the system friendlier and more accessible to the members of the deaf community. Signs are commonly represented using avatars or synthesized videos of a real human. The challenges of this task include the difficulty in creating realistic representations due to complex hand shapes and rapid arm movements.

### 5.1. Realistic Avatars

A common approach to sign language representation is the use of 3D avatars that with a high degree of accuracy and realism can reproduce facial expressions and body/hand movements in a way that represent signs understandable by deaf or hearing-impaired people. Balayn et al. in [99], developed a virtual communication agent for sign language to recognize Japanese sign language sentences from video recordings and synthesize sign language animations. Their system adopted a deep LSTM encoder-decoder network to translate sign language videos to spoken text, while a separate encoder-decoder network used as input the sign language glosses and extracted specific encodings, which were then used to synthesize the avatar motion. However, the network employed for the generation task does not have enough parameters to learn complete sentence expressions, lacking an attention module that could assist in learning longer-term dependencies. Shaikh et al. in [100], employed a system to generate sign animations from audio announcements in railway stations. At first, language rules and grammar was applied in the input text to transform it into a specific format. Then, inverse kinematics were applied to calculate the avatar target positions for each word and render the final video representation. Melchor et al. in [101], used a speech recognition system that translates Mexican spoken text into sign language. Then, the signs were represented through an avatar that was digitally animated on a mobile device. Uchida et al. in [102], developed an application to automatically produce sign language animations for sports games and was able to operate on live game broadcasts. A disadvantage of the application is that the delay time between the video occurrence and the video display is large.

Das et al. in [103], developed a 3D avatar to convert Indian text or speech into sign language. The input was translated to English and then to the corresponding Indian sign language using Natural Language Processing (NLP) rules and techniques. The final avatar movements were generated using a predefined sign vocabulary and Blender. A limitation of the system is that it was developed for a limited corpus and that the avatar had no facial expressions. Mehta et al. in [104], introduced a system in order to translate online videos into Indian Sign Language (ISL) and produce sign animations with a 3D cartoon-like avatar. The audio from the videos was captioned using NLP algorithms and mapped to signs that were finally rendered with the avatar. Nevertheless, due to the limited resources available for ISL, the performance of the system may degrade when dealing with complex grammatical structures and interactions. Patel et al. in [105], developed an application for animation generation. The input speech was recognised and translated with Google Cloud Speech Recognizer. Then, the translated text was converted to Hamburg notation system (HamNoSys) [106] and sign gesture markup language (SigML) [107] notations to effectively generate animations. Kumar et al. in [108,109] developed a mobile application to translate English text into ISL. HamNoSys was used for sign representation, SigML for its conversion to an XML file, and an avatar was employed to generate signs. A weakness of the developed system is that it struggles to represent complex animation and facial expressions of ISL signs. Moreover, the proposed system does not index the signs based on its context and this can cause confusion on directional signs that require different handling based on the context. Brock et al. in [110], adopted deep recurrent neural networks to generate 3D skeleton data from sign language videos. Subsequently, inverse kinematics were applied to calculate joints angles and positions that were mapped to a sign language avatar for animation synthesis.

### 5.2. Sign Language Production

Sign language production (SLP) has gained a lot of attention lately due to the huge advances in deep learning that allows the production of realistic signed videos. Sign language production techniques aim to replace the rigid body and facial features of an avatar with the natural features of a real human. To this end, these techniques usually receive as input sign language glosses and a reference image of a human and synthesize a signed video with the human performing signs in a more realistic way than the one that could have been achieved by an avatar.

Stoll et al. in [111], proposed an SLP method using a machine translation encoder-decoder network to translate spoken language into gloss sequences. Then, each gloss was assigned to a unique 2D skeleton pose, which were extracted from sign videos, normalized and aligned. Finally, a pose-guided generative adversarial network handled the skeleton pose sequence and a reference image to generate the gloss video. However, this methods fails to generate precise videos when the hand keypoints are not detected by the pose estimation method or the timing of the glosses is not predicted correctly. In their latest work, Stoll et al. in [112], used an improved architecture with additional components. The NMT network directly transforms spoken text to pose sequences, while a motion graph was adopted to generate 2D smooth skeletal poses. An improved generative adversarial network (GAN) was used in order to produce videos with higher resolution. The motion graph and the GAN modules improved significantly the quality of the generated videos. Stoll et al. in [113], adopted an auto-regressive gloss-to-pose network that can generate skeleton poses and velocities for each sign language gloss. In addition, a pose-to-video network generated the output video using a 2D-CNN along with a GAN. This approach resulted in smooth transitions between glosses and refined details on hand and finger shapes. Saunders et al. in [114], employed Transformers to automatically generate 3D human poses from spoken text using a multiple-level configuration. A text-to-gloss-to-pose (T2G2P) network with Transformer layers translated text sentences to sign language glosses and finally to 3D poses, while a text-to-pose (T2P) network directly transformed text into human poses. Furthermore, a progressive Transformer decoder was used to generate continuous and smooth human poses one frame at a time. Furthermore, the method achieved superior performance compared to NMT-based and GAN-based methods. Xiao et al. in [115] developed a bidirectional system for SLR and SLP. A deep RNN was used to jointly recognize sign language from input skeleton poses and generated skeleton sequences that were responsible to move an avatar or generate a signed video. The generated sequences were also used for SLR and improved the robustness of the system.

Cui et al. in [116], used a pose predictor network, which contains an LSTM and an autoencoder to generate the future human poses given a reference pose and the gloss label. Moreover, an image synthesis module accepted as input the current frame and the next pose to predict the next frame of the video using a U-Net based architecture with a CNN and an LSTM. Furthermore, it extracted regions of interest to improve details, such as the hands, which were crucial for generating high-quality sign language videos. This approach was able to synthesize realistic signs with naturally evolving hand shapes.

## 6. Applications

The advances in sign language capturing, recognition and representation have led to the development of several related applications. Each application can be compatible either with desktop computers or with android and iOS smartphones, as it is illustrated in Table 5. The majority of the methods use one or two CNN models integrated to their applications. The use of lightweight CNN models ensures the real-time performance of the applications.

Liang et al. in [117], introduced an automatic toolkit to recognize early stages of dementia among British Sign Language (BSL) users. Hand trajectory data, facial data and elbow distribution data were employed for feature extraction. The data were extracted using OpenPose and the dlib libraries. The final decision, whether the user was healthy or not, was taken by a CNN model. Zhou et al. in [118], created a Hong Kong sign language recognition platform, consisting of a mobile application and a Jetson Nano [130]. The mobile application was the front-end of the platform that preprocesses the sign language video. After the preprocessing, the video was transferred to the Jetson Nano that translates the video into spoken language, using a pre-trained deep learning model. Moreover, the authors created a Hong Kong sign language dataset for the purposes of the study. However, the method provides only word-level translation and predicts a relatively small vocabulary size. Furthermore, Ku et al. in [124], employed the 2d camera of the smartphone to record the signer. Hand skeleton information was extracted by OpenPose and a CNN model identified the meaning of the sign. The user could also choose to translate a pre-recorded video. However, very few gestures are recognised (three) and only finger positions are employed for feature extraction and not the entire hand. Moreover, the application does not run in real-time. On the other hand, Ozarkar et al. in [119], implemented a smartphone application consisting of three modules. The sound classification module detected and classified input sounds and alerted the user through vibrations. The gesture recognition module recognized the input Indian sign language video and converted it to natural language. In addition, the Multilingual Translation Module could either convert text to speech in different Indian regional languages or convert speech to text. Some limitations of the method are the performance degradation when more than one people appear in front of the camera, as well as the sensitivity of the sound classification module in noisy environments. Finally, Lee et al. in [126], described multiple technologies that could be integrated to a smartphone and ease the communication between speaking and hearing-impaired people. These technologies were: Text-To-Speech (TTS), Speech-To-Text (STT), Augmentative and Alternative Communication (AAC) and motion recognition.

Numerous educational oriented applications employing SLR have been also developed. These applications aim to help someone to learn or practice SL. Potamianos et al. in [125], presented a summary of the SL-ReDu project. The goal of the project was to teach the Greek sign language as a second language through recognition. The educational process was supported by self-monitoring and objective learning of the learners. Furthermore, a deep learning-based approach for isolated sign recognition of GSL was introduced. On the other hand, Joy et al. in [120], proposed a mobile application that could be used as a visual dictionary for children. It consisted of two modules: an object detection module and a word recognition module. The former enabled the user to select an object and the application displayed the corresponding sign. The latter took as input a picture of a text and it demonstrated the corresponding sign. However, the word recognition module is limited to translate a maximum number of 950 characters from a text. In addition, there are delays in loading sign animation videos due to the limited number of videos that can be stored on the mobile device. Moreover, Paudyal et al. in [121], designed a smartphone application that provides feedback to a sign language learner based on location, movement, orientation and hand-shape of his signs. A dataset was also created from 100 learners, for 25 American Sign Language (ASL) signs. However, the system does not perform continuous SLR. Schioppo et al. in [127], created a virtual environment for learning sign language, employing a virtual reality headset. A Leap Motion sensor was attached to the headset. The system was evaluated on the 26 letters of the alphabet in ASL. Luccio et al. in [122], employed an Elf Sandbot robot [131] to help people with hearing impairments to learn sign language. Two smartphone and tablet applications were also developed, with the first one controlling the movement of the robot and the second one taking a verbal or textual input of a word or sentence, translating it to sign language and demonstrating the corresponding video. Furthermore, Chaikaew et al. in [123], introduced an application that could help the communication of hearing-impaired people who want to learn the Thai sign language. The learners were able to choose the preferred vocabulary and practice with animation. Bansal et al. in [128], designed a game aiming to help Deaf children that lack continuous access to sign language, using only a high resolution camera and pose estimation software. The learner was asked to describe a scene and if the description was correct, he/she advanced to the next scene. Moreover, a dataset with RGB and depth features was created from adults with little experience with ASL. Nevertheless, the dataset consists of very few data to effectively train a deep learning model. Finally, Quandt et al. in [129], designed an avatar who served as the teacher of a virtual environment in order to teach introductory ASL to a novice signer. The users could also see a digital representation of their hands due to the usage of LEAP Motion. However, the system could not capture signs that involved touching a specific part of the body or signs that involved body part occlusion.

## 7. Conclusions and Future Directions

In this paper, the broad spectrum of AI technologies in the field of sign language is covered. Starting from sign language capturing methods for the collection of sign language data and moving on to sign language recognition and representation techniques for the identification and translation of sign language, this review highlights all important technologies for the construction of a complete AI-based sign language system. Additionally, it explores the in-between relations among the AI technologies and presents their advantages and challenges. Finally, it presents groundbreaking sign language applications that facilitate the communication between hearing-impaired and speaking people, as well as enable the social inclusion of hearing-impaired people in their everyday life. The aim of this review is to familiarize researchers with sign language technologies and assist them towards developing better approaches.

In the field of sign language capturing, it is essential to select an optimal sensor for capturing signs for a task that highly depends on various constraints (e.g., cost, speed, accuracy, etc.). For instance, wearable sensors (i.e., gloves) are expensive and capture only hand joints and arm movements, while in recognition applications, the user is required to use gloves. On the other hand, camera sensors, such as web or smartphone cameras, are inexpensive and capture the most substantial information, like the face and the body posture, which are crucial for sign language.

Concerning CSLR approaches, most of the existing works adopt 2D CNNs with temporal convolutional networks or recurrent neural networks that use video as input. In general, 2D methods have lower training complexity compared to 3D architectures and produce better CSLR performance. Moreover, it is experimentally shown that multi-modal architectures that utilize optical flow or human pose information, achieve slightly higher recognition rates than unimodal methods. In addition, CSLR performance on datasets with large vocabularies of more than 1000 words, such as Phoenix-2014, or datasets with unseen words on the test sets, such as CSL Split 2 and GSL SD, is far from perfect. Furthermore, ISLR methods have been extensively explored and have achieved high recognition rates on large-scale datasets. However, they are not suitable for real-life applications since they are trained to detect and classify isolated signs on pre-segmented videos.

Sign language translation methods have shown promising results although they are not exhaustively explored. The majority of the SLT methods adopt architectures from the field of neural machine translation and video captioning. These approaches are of great importance, since they translate sign language into spoken counterparts and can be used to facilitate the communication between the Deaf community and other groups. To this end, this research field requires additional attention from the research community.

Sign language representation approaches adopt either 3D avatars or video generation architectures. 3D animations require manual design of the movement and the position of each joint of the avatar, which is very time-consuming. In addition, it is extremely difficult to generate smooth and realistic animations of the fine grained movements that compose a sign, without the use of sophisticated motion capturing systems/technologies that employ multiple cameras and specialised wearable sensors. On the other hand, recent deep learning methods for sign language production have shown promising results at synthesizing sign language videos automatically. Besides, they can generate realistic videos using a reference image or video from a human, which are also preferable from the Deaf community instead of avatars.

Regarding the sign language applications, they are mostly developed to be integrated in a smartphone operating system and perform SL translation or recognition. A discrete category is the educational oriented applications, which are very useful for anyone with little or no knowledge of sign language. In order to create better and more easily accessible applications, the research should focus on the development of more robust and less computational expensive AI models, along with the further improvement of the existing software for integration of the AI models into smart devices.

**Figure 3:**
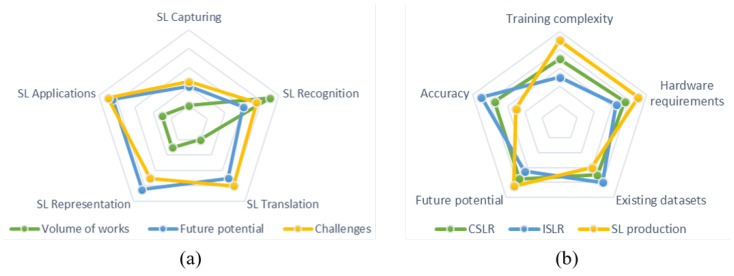
Radar charts showcasing the findings of this survey regarding (**a**) the literature methods for CSLR, ISLR and SLP and (**b**) the characteristics of each AI sign language technology.

Figure 3 is designed to provide objective and subjective comparisons of AI technologies and DNN architectures for sign language as seen from the perspective and the experience of the authors in the field. More specifically, Figure 3a presents and compares the characteristics of the different AI technologies for sign language. Volume of works is used to measure the number of published papers for each sign language technology and it is calculated based on the results of the query search in the databases. Challenges is used to subjectively measure the difficulty in accurately dealing with each sign language technology and it is based on the performance of the methods on the specific area. Finally, future potential is used to express the view of the authors on which sign language technology has the most potential to deliver future research works.

From the chart in Figure 3a, it can be seen that most existing works deal with sign language recognition, while sign language capturing and translation methods are still not thoroughly explored. It is strongly believed that these research areas should be explored more in future works. Furthermore, it is assumed that there is still great room for improvement for applications, especially mobile ones, that can assist the Deaf community. Regarding future directions, improvements can still be achieved in the accuracy of sign language recognition and production systems. In addition, advances should be made in the extraction of robust skeletal features, especially in the presence of occlusions, as well as in the realism of avatars. Finally, it is crucial to develop fast and robust sign language applications that can be integrated in the everyday life of hearing-impaired people and facilitate their communication with other people and services.

On the other hand, Figure 3b draws a comparison between various DNN architectures in terms of the performance of the proposed networks (Accuracy), hardware requirements for inference and training of the proposed networks (Hardware requirements), scope for improvement based on the performance gains and the volume of works (Future potential), computational complexity during training (Training complexity) and the number of recorded datasets that are currently available (Existing datasets). Except for the existing datasets, whose values are based on a search for publicly available datasets, all other metrics presented in the chart of Figure 3b are calculated based on the study of the review papers and the opinions and experience of the authors. As it can be observed, ISLR methods have high accuracy with small hardware requirements but such methods have been extensively explored resulting in limited future potential. On the other hand, CSLR and SLP methods have high hardware and training requirements, as well as demonstrate significant future potential as there is still great room for improvements in future research works.

## Figures and Tables

**Table 1:** Large-scale publicly available SLR datasets.

Datasets	Characteristics							
	Language	Signers	Classes	Video Instances	Resolution	Type	Modalities	Year
Phoenix-2014 [27]	German	9	1231	6841	210 × 260	CSLR	RGB	2014
CSL [30,31]	Chinese	50	178	25,000	1920 × 1080	CSLR	RGB, depth	2016
Phoenix-2014-T [28]	German	9	1231	8257	210 × 260	CSLR	RGB	2018
GRSL [15]	Greek	15	1500	4000	varying	CSLR	RGB, depth, skeleton	2020
BSL-1K [29]	British	40	1064	273,000	varying	CSLR	RGB	2020
GSL [17]	Greek	7	310	10,295	848 × 480	CSLR	RGB, depth	2021
CSL-500 [30,31]	Chinese	50	500	125,000	1920 × 1080	ISLR	RGB, depth	2016
MS-ASL [33]	American	222	1000	25,513	varying	ISLR	RGB	2019
WASL [34]	American	119	2000	21,013	varying	ISLR	RGB	2020
AUTSL [16]	Turkish	43	226	38,336	512 × 512	ISLR	RGB, depth	2020
KArSL [37]	Arabic	3	502	75,300	varying	ISLR	RGB, depth, skeleton	2021

**Table 2:** Performance comparison of CSLR approaches categorized by dataset measured in WER (%). The best performance for each dataset appears in bold.

Method	Input Modality	Dataset	Test Set (WER)
PL [58]	RGB	Phoenix-2014	40.6
RL [49]	RGB		38.3
Align-iOpt [55]	RGB		36.7
DenseTCN [57]	RGB		36.5
DPD [59]	RGB		34.5
CNN-1D-RNN [41]	RGB		34.4
Fully-Inception Networks [48]	RGB		31.3
SAN [47]	RGB		29.7
SFD [43]	RGB		25.3
CrossModal [52]	RGB		24.0
Fully-Conv-Net [42]	RGB		23.9
SLRGAN [53]	RGB		23.4
CNN-TEMP-RNN [39]	RGB+Optical flow		22.8
STMC [62]	RGB+Hands+Skeleton		**20.7**
DenseTCN [57]	RGB	CSL Split 1	14.3
Key-action [46]	RGB		9.1
Align-iOpt [55]	RGB		6.1
WIC-NGC [54]	RGB		5.1
DPD [59]	RGB		4.7
Fully-Conv-Net [42]	RGB		3.0
CrossModal [52]	RGB		2.4
SLRGAN [53]	RGB		**2.1**
STMC [62]	RGB+Hands+Skeleton		**2.1**
Key-action [46]	RGB	CSL Split 2	49.1
DenseTCN [57]	RGB		44.7
Align-iOpt [55]	RGB		32.7
STMC [62]	RGB+Hands+Skeleton		**28.6**
CrossModal [52]	RGB	GSL SI	3.52
SLRGAN [53]	RGB		**2.98**
CrossModal [52]	RGB	GSL SD	41.98
SLRGAN [53]	RGB		**37.11**

**Table 3:** Performance of ISLR methods on well-known datasets. The best performance for each dataset appears in bold.

Method	Dataset	Accuracy (%)
Konstantinidis et al. [74]	LSA64 [35]	98.09
Zhang et al. [70]		99.06
Konstantinidis et al. [75]		99.84
Gündüz et al. [77]		99.9
Huang et al. [89]	CSL-500 [31,32]	91.18
Zhang et al. [91]		96.7
Meng et al. [85]		97.36
Sarhan et al. [72]	IsoGD [36]	62.09
Zhang et al. [91]		63.78
Zhang et al. [70]		78.85
Rastgoo et al. [88]		86.1
Rastgoo et al. [63]		86.32

**Table 4:** Reported results on sign language translation on Phoenix-2014-T. The best performance appears in bold.

Method	Validation Set				Test Set			
	BLEU-1	BLEU-2	BLEU-3	BLEU-4	BLEU-1	BLEU-2	BLEU-3	BLEU-4
Sign2Gloss2Text [28]	42.88	30.30	23.02	18.40	43.29	30.39	22.82	18.13
MCT [97]	-	-	-	19.51	-	-	18.51
S2(G+T)-Transformer [96]	47.26	34.40	27.05	22.38	46.61	33.73	26.19	21.32
STMC-T [62]	**47.60**	**36.43**	**29.18**	**24.09**	**46.98**	**36.09**	**28.70**	**23.65**

**Table 5:** Characteristics of sign language applications.

Method	Operating System	Sign Language	Scenario
Liang et al. [117]	Windows desktop	British	Dementia screening
Zhou et al. [118]	iOS	Hong Kong	Translation
Ozarkar et al. [119]	Android	Indian	Translation
Joy et al. [120]	Android	Indian	Learning
Paudyal et al. [121]	Android	American	Learning
Luccio et al. [122]	Android	Multiple	Learning
Chaikaew et al. [123]	Android, iOS	Thai	Learning
Ku et al. [124]	-	American	Translation
Potamianos et al. [125]	-	Greek	Learning
Lee et al. [126]	-	Korean	Translation
Schioppo et al. [127]	-	American	Learning
Bansal et al. [128]	-	American	Learning
Quandt et al. [129]	-	American	Learning
